# Prey Preference of *Neoseiulus californicus* (McGregor) (Mesostigmata: Phytoseiidae) When Offered Two Strawberry Pests, *Scirtothrips dorsalis* Hood (Thysanoptera: Thripidae) and *Tetranychus urticae* Koch (Acari: Tetranychidae)

**DOI:** 10.3390/insects16111077

**Published:** 2025-10-22

**Authors:** Allan Busuulwa, Abigail Campos Gutiérrez, Sriyanka Lahiri

**Affiliations:** 1 Gulf Coast Research and Education Center, Entomology and Nematology Department, University of Florida, Wimauma, FL 33598, USA; 2 Campus Guácimo, Universidad EARTH, Mercedes, Limón P.O. Box 4442-1000, Costa Rica

**Keywords:** biological control, generalist predator, pest management, fruit crops, thrips, mites

## Abstract

Mites and thrips often infest strawberry crops across the globe, with devastating outcomes for growers. In fact, *Tetranychus urticae* Koch and *Scirtothrips dorsalis* Hood are major pests of strawberries in Florida, USA, often infesting plants at the same time. Chemical control is ineffective because both pests have developed resistance to many pesticides; therefore, many growers are presently utilizing predatory mites. Currently, *Amblyseius swirskii* Anthis-Henriot is released for *S. dorsalis* and *Phytoseiulus persimilis* Athias-Henriot for *T. urticae,* but this dual-release strategy is costly and can reduce effectiveness since *A. swirskii* can prey on *P. persimilis*. This study evaluated whether *Neoseiulus californicus* (McGregor) could provide a single practical alternative for managing both pests. We assessed its feeding preference in laboratory using no-choice and choice tests where an adult predator was offered different life stages of *T. urticae* and larvae of *S. dorsalis*. Results showed that in no-choice tests, *N. californicus* consumed more *T. urticae* eggs compared to *T. urticae* adults. Also, *S. dorsalis* larvae and *T. urticae* deutonymphs were consumed in similar proportions. In choice tests, feeding shifted toward mobile prey with higher consumption of *S. dorsalis* larvae and *T. urticae* deutonymphs and adults. These results demonstrate that *N. californicus* is a flexible predator with potential to control both pests and should be investigated further for its potential as a cost-effective tool in strawberry pest management.

## Simple Summary

Strawberry production in Florida faces serious challenges from two major pests, the twospotted spider mite (*Tetranychus urticae* Koch) and the chilli thrips (*Scirtothrips dorsalis* Hood), which often attack the crop at the same time. Growers usually rely on chemical sprays, but both pests have developed resistance to many commonly used products, leaving growers with limited options. As a result, strawberry growers have begun to release beneficial predatory mites. At present, *Amblyseius swirskii* Anthis-Henriot is used against chilli thrips, and *Phytoseiulus persimilis* Anthis-Henriot is used against twospotted spider mites. However, releasing two different species is costly, labor-intensive and has risks, since *A. swirskii* can also feed on *P. persimilis*, reducing its effectiveness. This study explored whether another predatory mite, *Neoseiulus californicus* (McGregor), could be used as a single solution for managing both pests. Our results show that *N. californicus* is a flexible feeder that can feed on chilli thrips larvae as well as all life stages of twospotted spider mites. These findings suggest that *N. californicus* may offer growers a more practical, and cost-effective, strategy for protecting strawberries from twospotted spider mites and chilli thrips.

## 1. Introduction

Predatory mites from the family Phytoseiidae are among the most important biological control agents used in many agricultural systems. Their effectiveness has been demonstrated across a range of crops, where they contribute significantly to biological control programs [1,2] by suppressing several economically important pests. Some of these pests include spider mites such as *Tetranychus urticae* Koch (Acari: Tetranychidae) [3], whiteflies (Hemiptera: Aleyrodidae) [4] and multiple thrips species (Thysanoptera: Thripidae) [5,6].

The ability of phytoseiid mites to suppress agricultural pests is fundamentally linked to their feeding behaviors, some of which have been the subject of extensive research, resulting in the development of a classification system of predatory mites based on their lifestyles [1,2,7]. This categorization has proven valuable for biological control programs because it provides a framework for selecting the most appropriate predatory mite for a given pest and crop.

Results from of this classification system have shown that although some predatory mites exhibit a high degree of specialization, many are generalist in their feeding behavior. A well-known example of a specialist predatory mite is *Phytoseiulus persimilis* Athias-Henriot (Mesostigmata: Phytoseiidae), which primarily feeds on spider mites [8,9], while *Neoseiulus californicus* (McGregor) (Mesostigmata: Phytoseiidae), once considered a specialist for spider mites, has since been shown to display more generalist characteristics [10], with the capability to feed on many thrips species [11,12].

The ability of *N. californicus* to feed on both *T. urticae* and thrips makes it a valuable predator in biological control programs where these pests occur simultaneously. This is particularly relevant in strawberry production in Florida, where strawberries face persistent pressure from *T. urticae* and the invasive *Scirtothrips dorsalis* Hood (Thysanoptera: Thripidae) [13]. The combined feeding damage caused by these two pests can significantly reduce yields, leading to major economic losses for many growers. Yield loss as a result of pest damage is critical in Florida, where strawberries are a high-value specialty crop and are a central part of the state’s agricultural industry [13]. Given that strawberry production in Florida is restricted to a short window (October to March), pest outbreaks can rapidly lower marketable yields and increase management costs. This challenge is even more pronounced in organic strawberry production, where growers have limited options for effectively managing pests.

In Florida, the generalist predatory mites *Amblyseius swirskii* Athias-Henriot and *Neoseiulus cucumeris* (Oudemans) (Mesostigmata: Phytoseiidae) have been reported to feed on and suppress populations of *S. dorsalis* [14]. Of the two, *A. swirskii* has proven to be more effective, providing greater suppression of *S. dorsalis* than *N. cucumeris*. The popularity of *A. swirskii* has also been driven by its wide commercial availability [15], making it a practical option for many growers. Furthermore, *A. swirskii* has been shown to successfully reduce *S. dorsalis* populations on strawberries under both field [5] and greenhouse conditions [16].

However, *A. swirskii* is not an effective predator of *T. urticae* [17]. This is because its foraging ability is hindered by the dense webbing produced by spider mites [18]. This limitation makes it unsuitable for the dual management of *S. dorsalis* and *T. urticae*. On the other hand, combining a spider mite specialist such as *P. persimilis* with a generalist like *A. swirskii* wouldn’t be practical given that it would result in intraguild predation on *P. persimilis*, leading to reduced control of *T. urticae* [19] and increased costs of production.

To this end, *N. californicus* has considerable potential for the dual management of *S. dorsalis* and *T. urticae* in both greenhouse and field settings, given its demonstrated ability to feed on and suppress populations of *T. urticae* and many other thrips species. However, before *N. californicus* can be recommended for widespread use against *S. dorsalis*, research is needed to clarify its prey preference when both *T. urticae* and *S. dorsalis* occur simultaneously. This study contributes to that effort by evaluating the prey preference of *N. californicus* when presented with *S. dorsalis*, *T. urticae*, or both. The findings from this work provide a foundation for assessing the suitability of *N. californicus* as a dual-purpose predator for the management of *S. dorsalis*, and *T. urticae* in Florida strawberry production.

## 2. Materials and Methods

### 2.1. Pre-Experimental Procedure

(i) Strawberry plant rearing

Strawberry plants (*Fragaria* × *ananassa*, Rosaceae, cv. Brilliance) were grown singly in 14 × 15 cm plastic pots filled with general-purpose growing medium (ProMix BX, Littleton, MA, USA) in the greenhouse facility at the University of Florida, Gulf Coast Research and Education Center, Wimauma, Florida. The greenhouse was set to follow ambient outdoor environmental conditions (temperature and humidity), which are appropriate for strawberry cultivation in Florida (October to March). Irrigation was done twice weekly with a nutrient solution prepared by mixing a balanced 20-20-20 all-purpose fertilizer (J R Peters Classic, Allentown, PA, USA) in water. The plants were kept under these conditions for a period of four weeks before being used in the experimental trials.

(ii) Predatory mite rearing

*Neoseiulus californicus* used to establish experimental colonies was obtained from Koppert Biological Systems (Howell, MI, USA). Laboratory colonies were initiated and maintained following the methodology described by Busuulwa et al. (2024) [20]. In brief, approximately 500 female *N. californicus* were transferred onto rearing arena using a fine paintbrush. The rearing arena was assembled using a plastic rectangular dish pan (35.6 × 29 × 12 cm; Greenbrier International, Inc., Chesapeake, VA, USA) filled to half its depth with distilled water. A large multipurpose sponge (19 × 14 × 2.5 cm; QEP, Boca Raton, FL, USA) was placed inside the pan as a support base, and a black flexible polystyrene board (12 × 8 cm; MEGA Format, Brooklyn, NY, USA) was placed on top to serve as the rearing surface. The perimeter of the board was lined with moist, sterile cotton (Fisher Scientific, Bridgewater, NJ, USA) to prevent mites from escaping. Oviposition structures like those described by Busuulwa et al. (2024) [20] were made and placed onto the arena to facilitate predatory mite oviposition.

To maintain the colonies, approximately 300–400 individual prey were provided every 48 h by placing a heavily infested leaf containing all life stages of *T. urticae* onto the arena. In addition, both first- and second-instar larvae of *S. dorsalis* were provided at the same time by brushing approximately 200 larvae onto the arena using a fine paintbrush. This approach was used to allow the predators to acclimate to the new prey species and ensure they readily recognized them as a food source [21]. The rearing arenas were then transferred to a growth chamber, maintained at 25 ± 1 °C and 70 ± 5% Relative Humidity and under a 14:10 h Light:Dark photoperiod.

*Scirtothrips dorsalis* and *T. urticae* used as food sources for the *N. californicus* in the experiments were obtained from established laboratory colonies. The *S. dorsalis* colony was maintained on cotton plants (*Gossypium hirsutum* L. (Malvales: Malvaceae)) inside a controlled-environment growth room set at 25 ± 1 °C, 65 ± 5% RH, and a 14:10 L:D. Colonies of *T. urticae* were maintained on bean plants (*Phaseolus vulgaris* L. (Fabales: Fabaceae)) under the same environmental conditions.

To obtain age-synchronized *N. californicus* for the bioassays, 300 gravid females were randomly selected from the main colony and placed on newly prepared rearing arenas to oviposit, after which point the females were removed from the arenas after 24 h. The arena containing the eggs were transferred into a controlled environment chamber set at 26 ± 1 °C, 70 ± 5% RH, and a 14:10 h L:D cycle. Upon hatching, the predatory mite nymphs were provisioned with the same prey items as those provided to the primary colony. Fresh prey was provided every 48 h until the predators reached maturity, which typically occurred eight days after hatching. The resulting age synchronized cohort of eight-day-old predatory mites was then used for all subsequent experiments.

### 2.2. Experimental Procedures

Prey preference of adult *N. californicus* was evaluated using randomized choice and no-choice experimental designs. In the no-choice experiments, each arena contained a single prey type (Table 1). One female from the age-synchronized *N. californicus* colony was introduced into each arena. The predation arenas were designed based off those described by Helle and Sabelis (1985) [22]. Essentially, the arenas were constructed by placing a detached strawberry leaf on a compressed wad of sterile cotton within a Petri dish containing distilled water. The strawberry leaf margins were lined with moist cotton to prevent both prey and predator from escaping. The arenas were then transferred into a growth chamber set at 26 ± 1 °C, 70 ± 5% RH, and 14:10 h L:D. The number of prey consumed from each arena was recorded every 24 h for a total of 72 h. The 72-h mark was chosen because in some prey types, the predators had consumed all the provided prey by 72 h. The consumed prey in each arena were not replenished during the experiment. Each prey item was tested with 15 replicates, and the experimental set up was repeated a second time (two trials).

In the choice experiments, arenas were prepared as described above, with all four prey types presented together in a single arena. A single female *N. californicus* from the age-synchronized colony was introduced and allowed to forage freely among prey types for 72 h. The arenas were maintained in a growth chamber under the same conditions as the no-choice experiment. Prey consumption for each prey type was recorded at 24-h intervals. This experiment also included 15 replicates and was repeated twice. Only first- and second-instar larvae of *S. dorsalis* were provided as food source to *N. californicus* because just like many other predatory mite species, *N. californicus* has been shown to prefer these stages. This preference is primarily because early instars exhibit fewer defensive behaviors, making them more susceptible to predation [11,12,14,17,20].

### 2.3. Statistical Analysis

Generalized Linear Mixed Effects Models (GLMMs) with a beta-binomial error distribution and logit link function were used to assess prey preference of *N. californicus* in choice experiments and to identify the most frequently consumed prey in the no-choice experiments. For both experiments, models were fitted using the *glmmTMB* package [23] with the proportion of prey consumed as the response variable. The fixed effects included prey provided, duration of the experiment and their respective interactions.

To account for the fact that the experiments included repeated measures, random effects were specified in the model with replicates nested inside the trials. The proportion of prey consumed was calculated as the number of prey consumed divided by the total number of prey provided at the beginning of each observation period. Furthermore, prior to modeling, observations in which the predator was absent during the first 24 h were removed from the dataset.

To ensure that the models adequately captured the structure of the data, the *DHARMa* package [24] was used to evaluate the fit and assumptions of the model. Simulated residuals were generated to assess dispersion, zero inflation, and deviations from the expected distribution. Residual plots were visually inspected for systematic patterns, and statistical tests implemented in *DHARMa* were applied to detect overdispersion and temporal autocorrelation.

Subsequently, analysis of deviance was conducted using the *Anova* function implemented in the *car* package [25] with *type II* Wald chi-square (χ^2^) tests. When significant differences were found, linear contrasts were carried out using the *emmeans* package [26], with a Tukey’s adjustment (Tukey’s HSD test, *p* < 0.05) applied to correct for multiple comparisons. All visualizations were created using the *ggplot2* package version 3.5.2 [27], and all analyses were conducted in R version 4.5.1 [28].

### 2.4. Model Diagnostic Results

#### 2.4.1. No-Choice Experiment

Model diagnostics using the *DHARMa* package revealed no violations of model assumptions. The Kolmogorov–Smirnov test (KS) for uniformity (*p* = 0.1916), dispersion test (*p* = 0.648), and outlier test (*p* = 0.64) showed no significant departures from expectations. The QQ plot of simulated residuals and the residuals versus predicted plot indicated no systematic patterns, supporting the adequacy of the model fit. Examination of the variance components showed that most random variation occurred among replicates within trials (variance = 0.315, s = 0.562) rather than between trials (variance = 0.107, s = 0.327), therefore indicating greater variability in prey consumption within trials than between trials.

#### 2.4.2. Choice Experiment

Similarly to the no-choice experiments, *DHARMa* residual diagnostics showed that the model met its assumptions. The QQ plot indicated a strong match between observed and expected values, with the KS showing no significant deviation from uniformity (*p* = 0.129). The dispersion test found no evidence of overdispersion or under dispersion (*p* = 0.72), and the outlier test detected no significant outliers (*p* = 0.98). Residuals were evenly distributed across predicted values with no apparent trends or heteroscedasticity. These observations indicated that the model fit well with the data. Additionally, the variance components of random effect showed that most random variation was among replicates within each trial (variance = 0.04, s = 0.12) compared to between trials (variance = 0.0042, s = 0.065). This meant that there was greater variability observed among replicates than between trials.

## 3. Results

### 3.1. No-Choice Experiment

Analysis of deviance showed significant effects of prey provided (χ^2^ = 52.50, df = 3, *p* < 0.001) and duration of the experiment (χ^2^ = 17.38, df = 2, *p* < 0.001) on prey consumption. The interaction between prey provided and duration of the experiment was also significant (χ^2^ = 31.12, df = 6, *p* < 0.001), showing that the consumption of prey provided varied across experimental durations.

**Figure 1:**
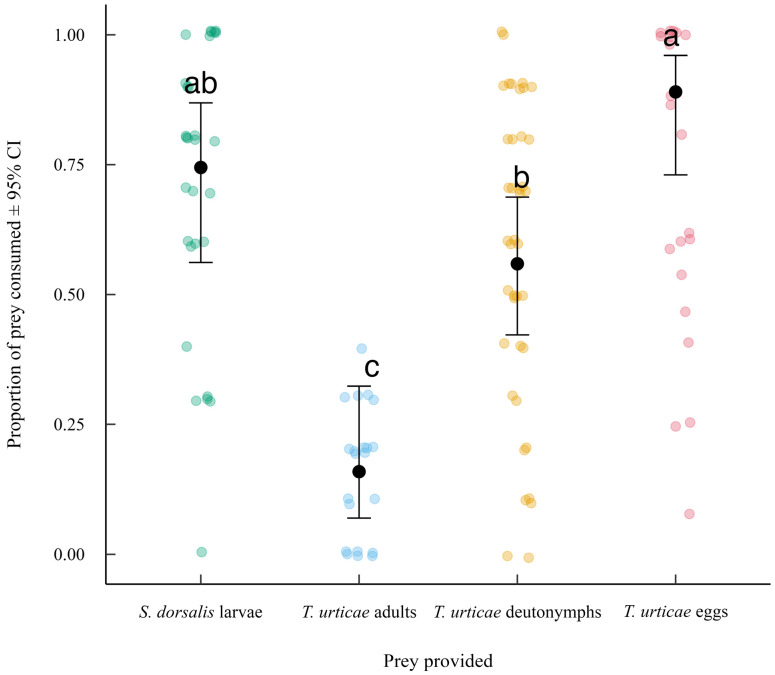
Mean proportion of prey consumed (±95% CI) by *N. californicus* in the no-choice experiment averaged across all experimental durations. Black dots represent estimated marginal means from the beta-binomial GLMM, and colored points show raw data prior to modeling. Different letters indicate significant differences among prey provided based on Tukey-adjusted comparisons (α = 0.05).

When averaged across experimental durations, post hoc Tukey-adjusted comparisons showed that *T. urticae* eggs had the highest proportion consumed, which was significantly greater than *T. urticae* adults and deutonymphs. There were no differences between the proportion of *Scirtothrips dorsalis* larvae, *T. urticae* eggs, and deutonymphs consumed by a single *N. californicus* female. Predator consumption of *T. urticae* deutonymphs was greater than that of *T. urticae* adults but lower than that of *T. urticae* eggs (Figure 1).

**Figure 2:**
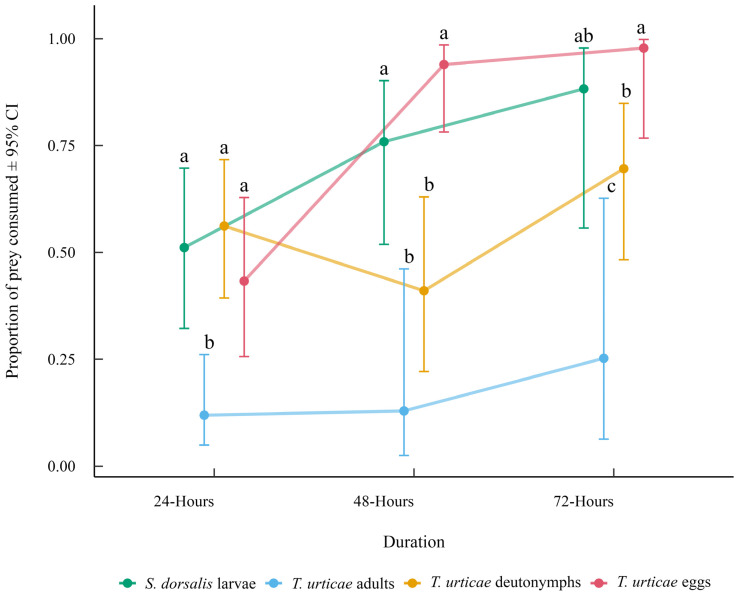
Mean proportion of prey consumed (±95% CI) by *N. californicus* in the no-choice experiment for each prey provided across experimental durations. Comparisons were made between prey types within each time point. Different letters indicate significant differences among prey types at the same duration based on Tukey-adjusted comparisons (α = 0.05).

The significant interaction between prey provided and experimental duration indicated that prey consumption patterns changed over time (Figure 2). At 24 h, *T. urticae* eggs, *S. dorsalis* larvae, and *T. urticae* deutonymphs were consumed in similar proportions, all higher than *T. urticae* adult consumption. By 48 h, *T. urticae* eggs and *S. dorsalis* larvae were consumed in higher proportions than *T. urticae* deutonymphs and *T. urticae* adults. At 72 h, *T. urticae* eggs remained the most consumed prey, followed by *S. dorsalis* larvae and *T. urticae* deutonymphs, with *T. urticae* adults consistently the least consumed at all durations.

### 3.2. Choice Experiments

The chi-square test conducted to assess whether the type of prey provided, and the duration of the experiment influenced the proportion of prey consumed by *N. californicus* showed that prey type had a significant effect (χ^2^ = 81.066, df = 3, *p* < 0.001) on the proportion of prey consumed. The duration of the experiment also had a significant effect on prey consumption (χ^2^ = 40.625, df = 2, *p* < 0.001). Additionally, the two-way interaction between prey type and experimental duration was significant (χ^2^ = 111.187, df = 6, *p* < 0.001). This meant that *N. californicus*’ s preference for a particular prey type changed depending on when observations were made.

**Figure 3:**
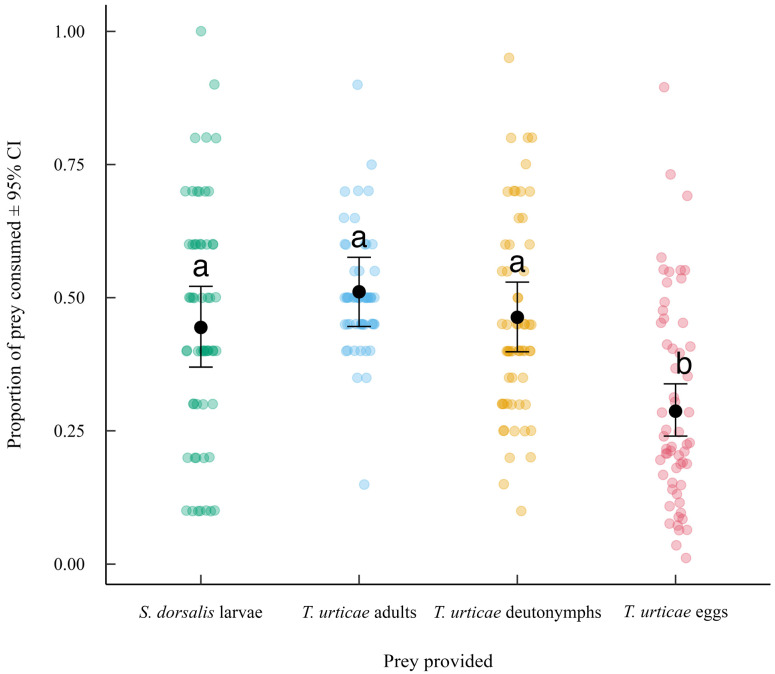
Mean proportion of prey consumed (±95% CI) by *N. californicus* in the choice experiment averaged across all experimental durations. Black dots represent estimated marginal means from the beta-binomial GLMM, and colored points show raw data prior to modeling. Different letters indicate significant differences among prey provided based on Tukey-adjusted comparisons (α = 0.05).

Results from the post hoc analysis (Tukey’s HSD test, *p* < 0.05) performed by averaging over experimental duration showed that the proportion of prey consumed by *N. californicus* varied among prey provided (Figure 3). *Scirtothrips dorsalis* larvae, *T. urticae* deutonymphs and *T. urticae* adults were consumed in higher proportions than *T. urticae* eggs, with no significant differences among the three mobile stages. These results showed that while the *N. californicus* readily consumed all mobile life stages, *T. urticae* eggs were the least preferred overall.

**Figure 4:**
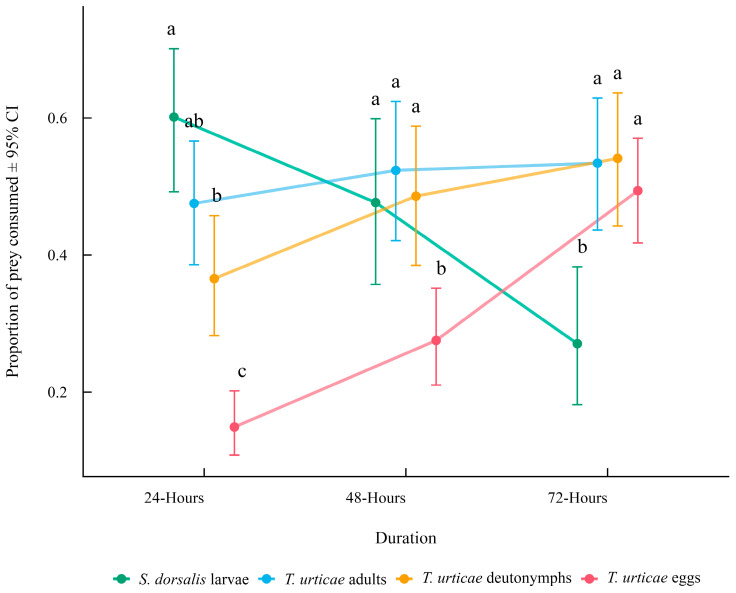
Mean proportion of prey consumed (±95% CI) by *N. californicus* in the choice experiments for each prey provided across experimental durations. Comparisons were made between prey provided within each time (duration) point. Different letters indicate significant differences in the proportion of prey consumed at the same duration based on Tukey-adjusted pairwise comparisons (α = 0.05).

The significant prey provided × experimental duration interaction showed that the predator’s preferences changed over time (Figure 4). At 24 h, *S. dorsalis* larvae were consumed most frequently and *T. urticae* eggs were the least consumed prey. By 48 h, consumption of *S. dorsalis* larvae, *T. urticae* deutonymphs and *T. urticae* adult stages was similar, with *T. urticae* eggs still consumed less often. At 72 h, *T. urticae* egg consumption increased to levels comparable with *T. urticae* adults and *T. urticae* deutonymphs, while *S. dorsalis* larval consumption declined.

## 4. Discussion

Understanding prey preference of a predator is essential when selecting a predator for use in biological control programs with multiple pests. We investigated whether *N. californicus* could feed on both *S. dorsalis* and *T. urticae* when presented individually or together. Results from this study indicate that *N. californicus* is a flexible predator when presented with *S. dorsalis* and *T. urticae*. In the no-choice experiments, *N. californicus* consumed more *T. urticae* eggs and the fewest *T. urticae* adults, with *S. dorsalis* larvae and *T. urticae* deutonymphs consumed equally. When prey were offered simultaneously, prey consumption shifted toward mobile prey stages. *Neoseiulus californicus* fed more on *S. dorsalis* larvae and *T. urticae* deutonymphs and adults, while *T. urticae* eggs were consumed less frequently. The predation patterns observed in this study suggest that *N. californicus* can feed on both *S. dorsalis* and *T. urticae*, but with a clear tendency to prefer mobile stages of both prey.

*Neoseiulus californicus* is classified as a Type II selective generalist predator of tetranychid mites [1,7] and has been extensively used to manage *T. urticae* [10]. However, Croft et al. (1998) [10] questioned its classification as a Type II, highlighting that it also exhibited some characteristic traits of Type III generalist predators. *Neoseiulus californicus* has been reported to feed on various thrips species [11,12,29] and pollen [30,31,32,33], a characteristic well known for Type III predators. McMurtry and Croft (1997) [1] noted that predator lifestyle types do not have clear boundaries, suggesting that some predatory mites could span more than one category. *N. californicus* may be such a predator given its diverse prey types. Depending on which prey are available, this species may shift from Type II to Type III and back to Type II.

Our observations in the no-choice experiments were consistent with the ecology of *N. californicus*, where the predator consumed more eggs of *T. urticae* compared to *T. urticae* adults. Prey consumption followed the order *T. urticae* eggs > *S. dorsalis* larvae > *T. urticae* deutonymphs > *T. urticae* adults, a pattern likely driven by differences in prey size. *Scirtothrips dorsalis* larvae are among the smallest larvae compared to those of other strawberry thrips pests such as *Frankliniella occidentalis* Pergande, *Frankliniella schultzei* Trybom, and *Frankliniella bispinosa* Morgan (Thysanoptera: Thripidae) and can even be smaller than adult *T. urticae* (personal observation). Additionally, it is known that when only one prey stage is available, predators tend to select the easiest item to capture and process. *Tetranychus urticae* eggs are immobile and lack defensive behaviors, which increases the predator’s encounter and attack success while reducing handling time. As a result, eggs become the default in no-choice experiments, even if their nutritional value is lower compared with mobile stages [34].

When multiple prey were provided in the choice experiments. *N. californicus* preferred feeding on mobile stages, especially *S. dorsalis* larvae and *T. urticae* deutonymphs and adults than *T. urticae* eggs. This shift in prey preference closely aligns with the optimal foraging theory, which predicts that predators maximize net energy gain by selecting prey that yield more energy per unit handling time [35]. Mobile thrips larvae and *T. urticae* deutonymphs and adults are generally more nutrient-dense compared to eggs; therefore, once alternatives are available, their higher net return often outweighs the added handling costs [36,37]. This kind of negative prey switching, where preference for prey types (especially eggs) declines as they become more abundant, has been reported for many generalist predators [37]. This is because in a natural setting, many generalist phytoseiid mites prefer to mix their diets to increase and balance nutrient uptake [22,38].

Specifically, it has been observed that *N. californicus* and *A. swirskii* tend to favor mobile stages when different prey stages are provided [32,39]. Another explanation for this shift in preference is that larvae, deutonymphs and adults are very active, which strengthens their detectability given that they produce stronger visual or tactile cues, increasing the probability of encountering the predatory mite [37,40]. Additionally, in predator–prey dynamics, prey profitability often depends not only on energy gain and handling time, but also on the predator’s ability to overcome defenses that generally increase with prey body size [41]. Sabelis and van Rijin (1997) [42] emphasized the importance of thrips size as a key factor in thrips predation by predatory mites. They noted that in practice, predation is most likely when prey fall within the predator’s manageable size window. Therefore, smaller thrips stages are preyed upon more frequently than larger, better-defended prey stages such as adults. This explains why in a multi-prey environment, smaller mobile prey stage were consumed in higher proportions compared to bigger mobile prey species.

In conclusion, our findings have important implications for strawberry pest management in Florida. They suggest that *N. californicus* could provide dual control of *S. dorsalis* and *T. urticae*, offering a more affordable option compared to the combined release of *A. swirskii* and *P. persimilis*, which is often too costly for growers. However, additional research is needed before *N. californicus* can be widely recommended. In particular, studies on its functional and numerical responses to mixed *S. dorsalis* and *T. urticae* are required, along with field experiments to evaluate how shared predation of *S. dorsalis* and *T. urticae* influences long-term population dynamics. These studies will clarify whether shared predation leads to apparent competition or other indirect interactions that could affect pest suppression outcomes.

## Figures and Tables

**Table 1:** Prey types and quantities provided to adult *Neoseiulus californicus* in the choice and no-choice experiments.

Prey Provided	Number of Prey Provided	
	Choice	No Choice
*Tetranychus urticae* eggs	50	50
*Tetranychus urticae* deutonymphs	30	10
*Tetranychus urticae* adults	30	10
*Scirtothrips dorsalis* larvae	10	10

## Data Availability

The raw data supporting the conclusions of this article will be made available by the authors on request.
